# Miller’s Case of Retention of the Fœtus in Utero, Three Years and Ten Months after the Full Term of Utero-Gestation

**Published:** 1842-10-29

**Authors:** Thomas Miller

**Affiliations:** Professor of Anatomy in the Columbian Medical College


### MEDICAL EXAMINER.

NEW SERIES.

No. 44.] PHILADELPHIA, OCTOBER 29, 1842. [Vol. I.

*Case of Retention of the Fœtus in Utero, three years and ten months after the full term of Utero-Gestation.* By Thomas Miller, M. D., Professor of Anatomy in the Columbian Medical College.

To the Editors of the Medical Examiner.

Gentlemen,—At a late meeting of the Pathological Society of this city, a remarkable case was presented by Dr. Miller, a copy of which, agreeably to a resolution which was then passed, I have now the honor to enclose you for publication.

Your ob't servant,

W. P. Johnston, M. D.

*Secretary of the Pathological Society.*

*Washington, (D. C.) Oct.* 15, 1842.

The case which I am about to present to you, is one in which most of the bones of a full grown fœtus were found in the uterus of a female twenty-two years of age, who died three years and ten months after the full term of utero-gestation. Labour pains had come on at the proper season, but ceased in a short time without affecting the expulsion of the fœtus. For most of the facts detailed in the following observation, I am indebted to Drs. Sewall, Munding and Magruder—not having seen the patient myself, until called upon to assist in the autopsy.

Mrs. Gass, a German, married and the mother of three children, was taken with symptoms of labour, in November, 1838.—Up to this period, she had enjoyed good health, and nothing unusual, as far as we can learn, occurred during this pregnancy. Dr. W. B. Magruder being called, considered her in active, natural labour.

The patient learning this, informed Dr. M., that as the case was not so urgent as she had supposed, she would send for the German physician, whom she had engaged to attend her. Early the next morning, Dr. Joseph Munding, also a German, was summoned to the aid of the attending physician, who had remained with her the whole night. The result of the deliberation of these gentlemen will be best explained by the following extract from a note I rereceived from Dr. Munding on the subject:

"In November, 1838, I was called early in the morning to see Mrs. G., not having been acquainted with her situation beforehand; although I had formerly attended under similar circumstances. When I arrived at her dwelling, I found a man there, whose name I have forgotten, and who informed me that Mrs. G. had been in labour since about 10 o'clock the preceding evening. The pains being slight, I made an examination per vaginam, and found the os uteri closed, and but little relaxation of the soft parts; the pains rather subsiding, and a little sanguineous discharge. Believing these to be mere preliminary symptoms of labour, and that the woman required rest more than anything else, we directed an anodyne mixture, consisting of laudanum and lac fœtidum, a few doses of which made the pains subside completely. I left her at break of day, giving it as my opinion, that delivery would not take place for at least twelve hours. I called several times during the day and found no return of pains, nor any other symptoms of approaching labour, and the woman being feeble, I ordered rest and the use of an infusion of matricaria chamomilla, with directions to be sent for, if any symptoms of labour should make their appearance. I called the next day and found no alteration, except that the sanguineous discharge was mixed with mucus, resembling what is termed lochia; the motions of the child were perceptible for eight days from my first visit. During six weeks following, I saw her occasionally, and no symptoms of labour had returned, after which time I lost sight of the patient, &c. &c."

We could learn nothing definite, as to the condition of the woman, from the period when Dr. Munding discontinued his attendance, except that she continued in much the same state, till May, 1839, when Professor Sewall was called to attend her. The Professor states, that Mrs. G.'s case first came under his consideration in May, 1839, (nearly six months after Dr. Munding had given her up;) "at which time," he says, "she exhibited the appearance of a woman in the seventh month of pregnancy, though with less regularity in the form of the abdomen—the projection being mostly on one side of the median line. At my first visit, she informed me that she had long transcended the usual period of gestation. I found by pressing with the hand, I could discover the form of a fœtus with remarkable distinctness, through the walls of the abdomen, and particularly the form of the head, and could even invert the convexity of some of the bones of the cranium. She however complained of great tenderness, and could not endure strong pressure made upon the fœtus, without experiencing considerable suffering. Upon examining per vaginam, I found the os tincæ and neck of the uterus healthy in structure and without any unusual tenderness, though she had for many months been subject to a copious discharge of yellow opaque matter, the properties of which I had not an opportunity of ascertaining.

"Taking all the attendant circumstances into view, (though I had no means of acquiring a history of the case, except from the woman herself, and that in very broken English,) I was disposed to regard it as extra-uterine pregnancy, and it was my wish to dilate the os tincæ sufficiently to explore the cavity of the uterus, and ascertain whether the fœtus was extra-uterine, or in its appropriate place, and whether there was an imperfection existing in the walls of the uterus, or communication between its cavity and that of the abdomen; but finding Mrs. G. and her husband averse to a consultation or any treatment except some palliative course, I contented myself by prescribing the tincture of iodine internally and the sulphate of zinc as an injection per vaginam.

"After a few occasional visits during the months of May, June and July, 1839, in my absence of several weeks from the city, she was removed from my charge, and I did not see her again until March, 1842, when I found her much wasted in flesh and strength, and labouring under hectic with a copious corrosive discharge from the vagina, which was extremely offensive. At this time, she informed me that for several months, she had occasionally discharged small bones and pieces of bones, by the vagina, some of which she had preserved. These I found to be fœtal bones, and composed of the metacarpus and phalanges, with several vertebræ and parts of vertebræ. Some of these bones were discharged during my late attendance and in the interval of my visits.

"My last visit, I find, was made in June, 1842, and of course I had no opportunity of learning anything of the closing history of the case; but taking all the circumstances which came under my observation, in connection with the facts disclosed upon your examination after death, is it not possible that if the state of the case could have been fully ascertained, at the time I first saw her, we should have found a portion of the walls of the uterus wanting, with the margin of the remaining portion attached to the parietes of the abdomen; so that, while the fœtus was in the cavity of the uterus, it lay in contact with the walls of the abdomen? If this were so, we see at once how the uterus had lost its expulsive power, and why the fœtus was left to decay and to pass off as it did.

"At what period this devastation of the uterus took place, and the adhesion of its margin to the parietes of the abdomen, I have no means of ascertaining, since I am entirely unacquainted with the early history of the case," &c. &c.

After Dr. Sewall discontinued his attendance, the case fell to the charge of Dr. W. B. Magruder, from whom I learn that there was no material variation in the symptoms: She continued to emaciate and lose strength, with occasionally violent pains, resembling dysmenorrhagic pains, which were attended with a more profuse discharge of the fluid above described by Prof. Sewall, tinged with blood. While Dr. Sewall was attending her, he informs me that the discharge was also occasionally tinged with blood. The bones of the fœtus continued passing now, both by the vagina and rectum. This poor woman continued going about and attending to her usual avocations, until within six weeks of her death, which occurred on the 25th of September, 1842. On the following morning, twenty-four hours after her death, the autopsy was performed in the presence of Doctors Magruder, Cuyler, and McClery.

*Autopsy.*—Body much emaciated; extremities œdematous; the abdominal tumour in the median line of the body spherical and as large as the uterus at seven months of pregnancy: the fœtal bones readily felt through the parietes of the abdomen; a very fœtid exhalation from the body; symptoms of incipient putrefaction about the abdomen. An incision in the course of the linea alba displayed the distended uterus adhering firmly to the surrounding parts, so much so, that it was only by close dissection that we were enabled to separate them, though this was done without opening either of the cavities of the surrounding organs, or destroying the integrity of the uterus itself. The parts to which it most firmly adhered, were the anterior parietes of the abdomen, the small intestines, omentum, which was much thickened, the colon and cœcum on the right side, the rectum *posteriorly*. Most of the abdominal viscera were agglutinated by the adherence of their serous surfaces and by false membrane. A number of distinct cysts, containing a yellow, semi-transparent, mucilaginous fluid, were in the neighborhood of the cœcum. Two large abscesses in right iliac fossæ, containing, one about a gill, and the other more than half a pint of unhealthy pus. The bladder was nearly healthy and somewhat distended with urine. The uterus being opened, a quantity of fœtid gas escaped, and the disorganized remains of a child were discovered. Nothing could be defined except the bones—these blackened, with their cartilaginous extremities and processes removed, and some portions of them presenting a worm-eaten appearance, but in other respects natural. The body and fundus of the uterus were so changed as scarcely to be recognised; the walls thinned to less than one-fourth of an inch; the inner or mucous surface presented a greenish and ulcerated appearance. Externally, its whole periphery was studded with patches of organised lymph; here and there could be seen the imperfect remains of the muscular coat; the whole flabby and much softened from the progress of the disorganization. A fistulous opening, an inch in diameter, between the uterus and rectum. The neck of the uterus nearly healthy—that is to say, a rim of about one inch in length, more thickened than natural, firmer than could have been anticipated, and of normal colour; the opening through the neck an inch in diameter and dilatable. Mouth of the uterus distended posteriorly into a pouch, large enough to hold a gill; which was filled with small bones such as had already passed, mixed up with portions of the disorganized remains of the fœtus—as those were (the bones) which were found in the cavity of the uterus. The mouth was much thinned, its mucous membrane being in the same condition as that of the uterus. Vagina flabby, its color greenish."

In presenting to the Pathological Society the foregoing case, I have been actuated by a desire to preserve one of the rarest and most interesting cases it has ever been my fortune to meet with.

The history of this case is incomplete only in two periods, viz: as to the actual and specific condition of the patient during the period of pregnancy, to the time when Dr. Magruder was first called; and secondly, the interval from the cessation of Dr. Munding's visits to Prof. Sewall's first call.

Though the patient was not at either of these periods under the immediate charge of any medical gentleman, she was in both instances occasionally seen by Dr. Magruder, who attended other members of the family, and it is to be presumed that she was not in either instance actually suffering; otherwise she would have resorted to counsel.

As to her previous history, we learn that she was the offspring of healthy parents, though she was herself rather delicate, of a leuco-phlegmatic temperament. She was married and became a mother before she was fifteen years old.

It may be of interest to state, that one of her labours proved tedious and protracted; the placenta was retained, as will be seen in the case reported by Prof. Lindsley in the 19th volume of the American Journal of Medical Science, page 337. Whether the placenta was absorbed in this case or not, it is certain that the uterus recovered its healthy condition very soon; for we are informed by Dr. Munding, who attended her in two subsequent labours, that she proved pregnant in both instances, in six months after delivery, and did well after each labour. Whether this be the same case as that reported by Dr. Lindsley, I am unable to vouch with certainty. Some discrepancies exist between the cases, as to name, age, &c., which I am unable to reconcile. I state the circumstances upon the assurance of others.

It would now be interesting to inquire into the cause of the detention of the fœtus. These cases are of such rare occurrence, that we have scarcely a starting point. We do not, however, propose to undertake this task; all we design doing is, to call your attention to some of the facts as revealed by the autopsy, and leave it to you to solve the difficulty, if possible.

That there was a diseased condition of the uterus at or previous to the period of the commencement of labour, I have little doubt; and this disease, most probably of the peritoneal coat, had resulted in adherence of the uterus to the parietes of the abdomen. This opinion seems to be justified by the result of the autopsy. The second suggestion I will offer is, that the fœtus might have been enclosed in a cyst of so strong a texture, as to have resisted the expulsive power of the uterus in the early period of labour, and the expulsive power being once lost, and disease at the same time being established in the uterus, adhesion occurred, and its efforts afterwards were thus rendered abortive.

If the autopsy has been imperfect, inasmuch as it does not extend beyond its immediate object, viz: the discovery of the exact location of the fœtus and the condition of the uterus, we must plead as our apologies, *first*. The short time allowed for its performance; *secondly*. The condition of the body; and *thirdly*. We had no reason to believe that any other part of the body had any agency in the production of the difficulty which existed.

The uterus and fœtal bones obtained from the patient, who is the subject of the above observation, have been deposited in the museum of the Pathological Society.

